# Guiding of Cell Migration over Sloped Steps Using TiO_x_ Arrowhead Patterns

**DOI:** 10.3390/jfb17070323

**Published:** 2026-07-05

**Authors:** Yijun Cheng, Chang Liu, Stella W. Pang

**Affiliations:** 1 Department of Electrical Engineering, City University of Hong Kong, Kowloon, Hong Kong, China; 2 Centre for Biosystems, Neuroscience, and Nanotechnology, City University of Hong Kong, Kowloon, Hong Kong, China; 3 State Key Laboratory of Terahertz and Millimeter Waves, City University of Hong Kong, Kowloon, Hong Kong, China

**Keywords:** unidirectional migration, patterned titanium oxide, sloped steps, gratings, asymmetrical arrowheads

## Abstract

Cell migration is a fundamental biological process regulated by interactions between cells and extracellular matrix. Although topographical cues are known to influence cell behaviors, directional migration across three-dimensional (3D) sloped steps remains poorly understood. Here, 3D sloped steps with patterned TiO_x_ surfaces were fabricated to investigate topography-guided cell migration in complex 3D microenvironments. The ultrathin TiO_x_ layers were patterned along the bottom, sidewall, and top regions of the steps, providing continuous guidance during cell migration up or down the steps. MC3T3-E1 cells were confined to the patterned regions and exhibited contact-guided migration along the asymmetrical arrowhead patterns. Forward and reverse arrowheads were introduced to evaluate the effect of geometrical asymmetry on cell migration directionality. Forward arrowheads preferentially guided cells from the bottom to the top of steps, whereas reverse arrowheads promoted migration down the steps, demonstrating reversible control of cell migration direction through arrowhead orientation. Analysis of cell morphology revealed that ultrathin TiO_x_ topographies influenced lamellipodia orientation and cell adhesion, providing mechanistic insights into geometry-mediated control of cell migration direction. These findings demonstrate that guiding pattern asymmetry can be used to regulate the speed and directionality of cell migration across sloped steps, which can be applied to control cell migration behaviors on engineered 3D platforms.

## 1. Introduction

Cell migration is a fundamental biological process for tissue development, wound repair, immune regulation, and cancer progression [1,2,3]. Cell migration behavior is governed by dynamic interactions between intracellular activities and extracellular environmental cues [4,5,6,7,8]. The extracellular matrix (ECM) provides cells with guidance cues, including topography pattern, adjustable stiffness, chemical functionality, and surface energy [9,10,11,12]. Among these cues, surface topography has emerged as a key regulator of cell migration [13,14,15]. To achieve a deeper understanding and more precise control-directed cell migration, a variety of contact-guidance strategies have been developed. Symmetrical micropatterns, such as gratings, are commonly used to guide cell migration bidirectionally [2,16]. However, these patterns typically lead to migration along the patterns with frequent directional changes and limited long-term directional persistence. In contrast, asymmetrical micropatterns provide directional cues that promote cytoskeletal polarization and generate biased traction forces, facilitating unidirectional migration [17,18]. While previous studies have offered valuable insights into how cells interact with two-dimensional (2D) patterned surfaces, they primarily highlight cellular adaptation of shape and cytoskeletal organization within planar environments.

However, cells often encounter complex three-dimensional (3D) microenvironments in vivo, including sloped steps and various topographies, which are common in tissues such as bone, vasculature, and epithelial layers [19,20]. Numerous studies have investigated cell migration in 3D environments [21]. For example, human bone marrow stromal cell migration on anisotropic curved structures has been investigated. On substrates featuring orthogonally oriented convex and concave curvatures, migration speed was found to be direction-dependent, with cells preferentially aligning along trajectories that minimize membrane bending [22,23,24,25]. Microgroove-based topographies have been fabricated to control the migration of various cancer cell types, highlighting differences between cancerous and non-cancerous epithelial cells. Non-cancerous epithelial cells conformed to the 3D geometrical cues and migrated along the walls, whereas cancerous cells exhibited the distinctive behavior of moving up walls [26,27]. Ultra-smooth sinusoidal surfaces with modulations of curvature in all directions have been developed to monitor cell behaviors. Adherent cells tend to avoid convex regions during migration and preferentially occupy concave valleys [28]. Cells bend more significantly in regions of higher curvature, exhibiting reduced spreading and lower motility [29,30,31]. Additionally, barriers of different heights, combined with grating and arrowhead patterns in channels, have been used to guide cell migration. Taller barriers hinder vertical migration of MC3T3-E1 cells, whereas gratings and arrowheads facilitate it [32]. These existing 3D studies primarily focus on the effects of curvature or confinement on cell migration and morphology. While previous work has examined migration along vertical directions, surface patterns were limited to the bottom region while leaving slopes and tops of steps unpatterned. As a result, achieving precise directional control of cell migration across sloped steps remains a major challenge in biointerface engineering.

MC3T3-E1 cells possess the ability to differentiate into osteocytes and form calcified bone tissue in vitro. They also have a proliferation rate, matrix mineralization pattern, and alkaline phosphatase activity that are closely resemble those of primary osteoblasts. These characteristics make MC3T3-E1 cells a highly suitable and reliable model for conducting in vitro biomaterial testing, facilitating valuable insights into bone-related biomedical research. In this study, the well-characterized MC3T3-E1 cells were chosen to minimize biological variability. Future studies will investigate additional cell types to further explore the broader applicability of the platforms. PDMS sloped steps patterned with ultrathin TiO_x_ layers including symmetrical gratings and asymmetrical arrowheads were fabricated that provided directional cell migration cues. The primary biological question addressed in this work is how asymmetrical 3D topographical patterns could regulate cell migration direction during step crossing. Cells moving across the sloped steps were evaluated and the influence of the guiding patterns and slope angle on directional preference, probability of moving over steps, migration time, and speed were investigated. Our results show forward arrowheads promoted cell migration up the slope, whereas reverse arrowheads favored migration down the slope. Collectively, this work demonstrates that ultrathin TiO_x_ arrowhead patterns provided an effective approach to control unidirectional cell migration across slopes. Overall, to systematically investigate the combined effects of sloped surface and patterned guidance on cell migration, steps with two slope angles plus a control flat surface with a 0° angle, with each sloped step having two guiding pattern designs for unidirectional and bidirectional guidance plus a control surface without guidance, were developed to investigate slope- and guidance-dependent migration behaviors. The guiding patterns used in this study were designed to provide directional cell migration guidance. They were used to generate the fundamental information needed to understand cell–topography interactions at the cellular level, which can be applied to various cell types. The flat PDMS platforms with patterned TiO_x_ were used as the 0° control. These devices contained the same TiO_x_ patterns on flat surfaces, including grating and arrowhead configurations, but without sloped steps. Therefore, platforms on flat surfaces with 0° slope served as controls for evaluating how the introduction of 3D sloped geometry affected cell migration behavior. These devices allow for comparison between 2D planar contact-guided migration on a flat surface with 0° slope and step-crossing migration on 20° and 40° sloped 3D platforms.

## 2. Experiment and Methods

### 2.1. Fabrication Technology for TiO_x_ Patterns on Sloped PDMS Substrate

**Figure 1:**
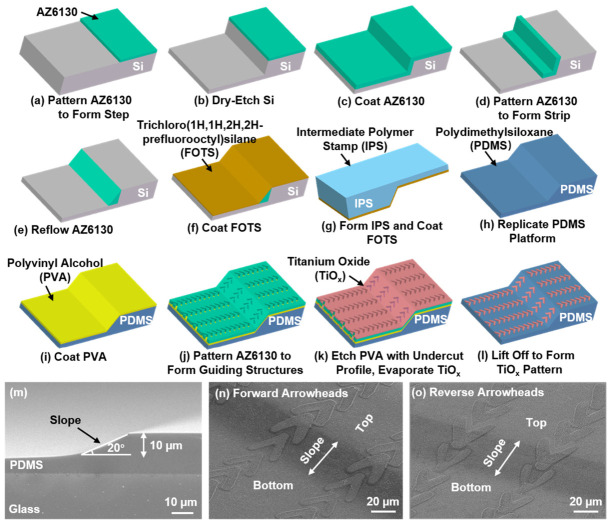
Fabrication technology and micrographs of patterned titanium oxide (TiO_x_) over sloped steps in polydimethylsiloxane (PDMS). (**a**,**b**) Photoresist AZ6130 spin-coated onto Si and patterned by photolithography, followed by dry etching to form vertical Si step. (**c**–**e**) AZ6130 photoresist was coated, patterned, and reflowed to form Si sloped steps. (**f**–**h**) Intermediate polymer stamp (IPS) was replicated from Si stamp using imprint technology. PDMS platform with sloped steps was formed using IPS. (**i**,**j**) Cell migration guiding patterns in photoresist were formed on PDMS platform coated with polyvinyl alcohol (PVA). (**k**,**l**) Undercut profile in AZ6130 was generated using O_2_ plasma, followed by TiO_x_ evaporation and liftoff to form guiding patterns. (**m**–**o**) Micrographs of sloped steps with forward (arrowhead tips pointed up slope) and reverse (arrowhead tips pointed down slope) arrowheads.

Figure 1a–l illustrate the fabrication technology for forming TiO_x_ patterns on sloped PDMS substrate. Firstly, Si substrate underwent an oxygen (O_2_) plasma treatment for 30 s, followed by AZ6130 photoresist coating, soft-baking at 105 °C for 5 min, exposure for 20 s, development for 40 s, and hard-baking at 120 °C for 10 min, as shown in Figure 1a. Dry etching was conducted using 138/11 sccm SF_6_/O_2_ gas flow at 28 mTorr with 600 W RF coil power and 14.8 W RF platen power for 3 min to form a 10 µm tall vertical step in Si, as shown in Figure 1b. Next, AZ6130 photoresist was coated onto the Si wafer with the vertical steps at 1000 rpm for 1 min, as shown in Figure 1c. The sample was patterned to form 20 µm wide strips and baked at 180 °C for 1 h to make photoresist strips reflow to form a 20° sloped Si surface, as shown in Figure 1d,e. Then the sloped surface was coated with trichloro(1H, 1H, 2H, 2H-perfluorooctyl)silane (FOTS) after an O_2_ plasma treatment. An intermediate polymer stamp (IPS) was replicated from the Si substrate with sloped steps under the imprint conditions at 150 °C and 40 bar for 2 min. A second FOTS layer was coated on the replicated IPS. Hard polydimethylsiloxane (hPDMS, base:cross-linker weight ratio = 1:1, Gelest, Morrisville, PA, USA) was spin-coated on a glass substrate and the replicated IPS was pressed onto the hPDMS layer. After curing at 80 °C for 4 h, the IPS was peeled away, and PDMS platforms with sloped steps were formed, as shown in Figure 1f–h.

A 6% (weight by volume) water-soluble polyvinyl alcohol (PVA, Invitrogen, Waltham, MA, USA) was spin-coated on the PDMS substrate with plasma treatment at 3000 rpm for 60 s and then baked on a hotplate at 105 °C for 10 min. AZ6130 photoresist with 4 μm thickness was spin-coated on the PVA film. The arrowhead pattern on sloped steps was formed using photolithography, as shown in Figure 1i,j. The sample was etched to form an undercut profile using reactive ion etching (RIE) (790 RIE system, Plasma-Thermal, St. Petersburg, FL, USA) with conditions of 20 sccm O_2_, 40 mTorr, 100 W, and 6 min, followed by 10 nm thick Ti deposition using an electron beam evaporator (ATS 500, HHV Advanced Technologies, West Sussex, UK), as shown in Figure 1k. Subsequently, the patterned TiO_x_ was formed after removing the PVA sacrificial layer via ultrasonication in DI water for 20 min and nitrogen drying, as shown in Figure 1l. Micrographs of the sloped steps with forward and reverse arrowheads are shown in Figure 1m–o. The purpose of the present platform design is to develop a biomimetic physical microenvironment in which cells encounter 3D topographical surfaces during migration. In vivo, cells frequently migrate across complex ECM environments consisting of local height variations, sloped interfaces, ridges, and step-like topographies. Such 3D microstructures with slopes are commonly found in bone remodeling sites, microfracture regions, tissue interfaces, and biomaterial implant surfaces, where osteoblast-like cells must actively traverse nonplanar topographies during migration. The sloped steps used in this study therefore serve as a simplified and well-controlled model for investigating how cells respond to topographies during migration along the vertical direction. The arrowhead patterns provide controlled anisotropic cues for studying how geometric asymmetry regulates migration directionality. This designed 3D platform allows for systematic investigation of cell–topography interactions beyond the 2D planar surfaces to mimic cell movements in all directions including the vertical orientation.

Sloped steps were 10 µm in height and slope angle was 20°. Arrowhead arms were 5 µm wide, 20 µm long, and 10 nm tall with 5 µm separation and an arrowhead tip angle of 45°. The arrowheads with pointed tips were designed to control the direction of cell migration. Two key parameters were considered: the arrowhead angle and the dimension of arrowhead arms. The tip angles were set to 45°, 90°, and 135°. When the tip angle was 90° or 135°, cells could migrate along the arrowhead arm direction or change direction at the tips. In contrast, when the tip angle was 45°, cells could form large cytoplasmic protrusions around the arrowhead tips and migrate along the arrowhead tip direction. In this study, no physical wall is needed to confine the cells on the patterns. Instead, an arrowhead pattern with a thin TiO_x_ layer was used to guide unidirectional cell migration. By generating a surface energy difference between the PDMS substrate and the TiO_x_ surface, the patterned arrowheads confined cell migration on the TiO_x_ patterns. Furthermore, the asymmetrical geometry of the arrowheads induced larger cytoplasmic protrusions along the arrowhead tips, resulting in directional cell migration along the tips.

### 2.2. Cell Culture and Seeding

MC3T3-E1 pre-osteoblastic cells were obtained from American type culture collection CRL-2594 (American type culture collection, Manassas, VA, USA) and were cultured in high-glucose Dulbecco’s modified eagle medium (DMEM, Invitrogen, Waltham, MA, USA). The DMEM medium was supplemented with 10% fetal bovine serum (FBS, Gibco, Waltham, MA, USA) and 2 mM alanyl-L-glutamine (GlutaMAX^TM^, Gibco, Waltham, MA, USA). Cells were incubated at 37 °C in the humidified atmosphere with 5% CO_2_. To maintain optimal cell motility and viability, the culture medium was refreshed every 2 days. Cells were passaged before reaching 80% confluency to maintain healthy growth conditions. The fabricated PDMS platforms with patterned TiO_x_ on sloped steps were attached to the bottom of the circular culture dishes using a thin layer of PDMS as an adhesive material. Prior to cell seeding, the PDMS substrates were washed with 70% alcohol and phosphate-buffered saline (PBS) twice. MC3T3-E1 cells were subsequently seeded uniformly onto the entire surface of the patterned platforms, allowing cells to freely settle on both the patterned and non-patterned regions. After seeding, cells were incubated for 6 h to ensure stable cell adhesion to the PDMS platforms prior to imaging. The culture medium was then replaced with a CO_2_-independent medium (Invitrogen, USA) supplemented with 10% FBS and 2 mM alanyl-L-glutamine for time-lapse imaging, which was performed to record cell migration dynamics. For quantitative analysis, only cells that were adhered to the patterned regions were included and tracked, whereas cells residing outside the patterned areas were excluded to ensure that all analyzed trajectories corresponded to cells in contact with the designed topographical features.

### 2.3. Time-Lapse Imaging and Data Analysis

Images were acquired every 5 min over a 16 h period using an upright optical microscope (Eclipse Ni-E, Nikon, Tokyo, Japan) equipped with a 20× objective lens. ImageJ software (1.48v, NIH, Bethesda, MD, USA) was used to analyze the migration trajectories and migration speeds of MC3T3-E1 cells. During imaging, cells were maintained in an incubation chamber at 37 °C. Only cells that remained viable, did not undergo division, and showed no interaction with neighboring cells throughout the 16 h imaging period were included in the analysis. The number of MC3T3-E1 cells migrating in all possible directions along the sloped PDMS platform was counted at the moment each cell contacted the sloped step boundary. Up slope time was measured as the duration from when a cell first contacted the base of the sloped step until its entire cell body reached the top, while down slope time was measured as the duration from initial contact with the top edge of the sloped step until the entire cell body reached the bottom of the step. Data was obtained from at least three independent experiments under identical conditions with the same cell culture environment, incubation time, and measurement methods. Statistical analysis was performed using one-way analysis of variance (ANOVA) followed by Tukey’s post hoc test to determine significant differences between groups.

### 2.4. Cell Imaging Using Scanning Electron Microscopy

After time-lapse imaging, the culture medium was removed and the platform was rinsed with 1× PBS for 5 min. The cells were then fixed with 4% paraformaldehyde (PFA, Sigma-Aldrich, St. Louis, MO, USA) for 15 min at 25 °C. The samples were sequentially washed with 1× and 0.25× PBS for 5 min each, followed by two rinses in DI water for 10 min each. Subsequently, the cells were dehydrated through a graded ethanol series (30%, 50%, 70%, 80%, 95%, and 100%) for 5 min at each concentration. The samples were then dried using a critical point dryer and sputter-coated with a thin gold layer to improve surface conductivity. High-resolution images of the fixed cells were acquired using a field-emission scanning electron microscope (SU5000 FE-SEM, Hitachi, Tokyo, Japan) operated at 10 kV. These images were used to investigate the interactions between MC3T3-E1 cells and the different patterned surfaces on sloped steps, providing insight into guided cell migration across stepped platforms.

## 3. Results

To systematically investigate the effects of patterned guidance and slope angle of step on cell migration behaviors, two guiding patterns and two slope angles were developed. First, flat PDMS surfaces with a 0° slope angle were patterned with TiO_x_ gratings for bidirectional guiding and arrowhead patterns for unidirectional guiding, and this served as a control group compared to the ones with sloped steps. These guiding patterns enabled evaluation of the distinct guidance characteristics of symmetrical gratings and asymmetrical arrowhead patterns on cell morphology, migration directionality, and migration speed.

The same guiding patterns were also integrated onto the 20° and 40° sloped steps to investigate how these guidance cues interact with cells on sloped steps. Since cells in physiological environments frequently encounter nonplanar and discontinuous topographies, the introduction of sloped steps allowed for the assessment of cell migration across steps with different angles.

Therefore, the designed experimental groups consisted of two major elements, and they were compared to control groups: (i) engineered patterns for unidirectional and bidirectional cell migration guidance and (ii) cell migration over steps with 20° and 40° slopes. This systematic approach enabled investigation of how guiding patterns and steps with slope angles regulate cell migration across platforms with nonplanar surfaces.

### 3.1. Pattern-Dependent Cell Migration Directionality on Flat PDMS Platforms with 0° Slope Angle

**Figure 2:**
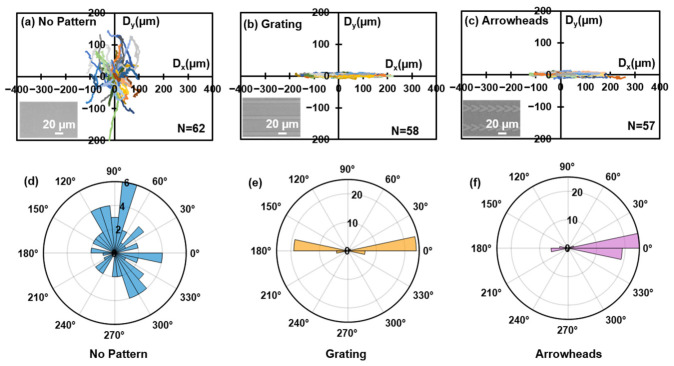
Migration trajectories of MC3T3-E1 cells on flat PDMS platforms with 0° slope angle with (**a**) no pattern, (**b**) grating, and (**c**) arrowheads. Gratings were 10 µm wide, had 40 µm spacing, and were 10 nm tall. Analysis of MC3T3-E1 cell migration directions showed (**d**) random directions on the surface without a pattern, (**e**) bidirectional cell migration on grating, and (**f**) unidirectional cell migration on arrowheads.

As a control group, cell migration behaviors on flat PDMS platforms with 0° slope angle and two different guiding patterns were investigated. Figure 2a–c illustrate migration trajectories of MC3T3-E1 cells on flat PDMS platforms with 0° slope angle and no guiding pattern, gratings, and arrowheads. On a grating pattern that was 10 µm wide, had 40 µm spacing, and was 10 nm tall, cells exhibited clear bidirectional migration along the grating orientation. Cells on an arrowhead pattern that had a 20 µm arm length, 40 µm spacing, and 10 nm height were constrained to migrating primarily along the arrowhead orientation, that is, unidirectionally.

Supplementary Figure S1a shows the cell migration speed, with V_x_ in the x-direction and V_y_ in the y-direction. The deviation angle θ represents the cell migration directionality on the platforms with 45° representing random migration direction. The measured deviation angles for no pattern, grating, and arrowheads are 45.9°, 11.8°, and 13.9°, respectively. On the surface without a pattern, cells moved with similar speeds in both the x- and y-directions, indicating they moved randomly without orientation preference. A smaller angle means cells were guided along the orientations of the gratings and arrowheads. The average cell migration speeds were measured to be 0.34 ± 0.09, 0.49 ± 0.11, and 0.43 ± 0.08 μm/min on flat PDMS platforms with 0° slope angle without a pattern, with gratings, and with arrowheads, respectively. Cell migration speed was faster on patterned TiO_x_ gratings and arrowheads, as shown in Supplementary Figure S1b.

To further investigate the unidirectionality of cell migration on flat PDMS platforms with 0° slope angle, the migration directions of MC3T3-E1 cells were calculated using the distribution angle between the instantaneous velocity vector and the x-axis, as shown in Figure 2d–f. These distributions were visualized using polar plots, where 360° was divided into thirty 12° sectors. The length of each radial axis represents the number of cells migrating within the corresponding angular range, facilitating direct comparison across different surfaces. On surfaces without guiding patterns, cells exhibited random migration directions. On gratings, migration was primarily bidirectional with cells moving along both directions of the gratings. On arrowhead patterns, cells displayed strongly unidirectional migration with cells moving along a single direction towards the tips of arrowheads.

### 3.2. Cell Migration Dynamics and Unidirectional Guidance Across Sloped Steps

How cells dynamically migrate on sloped surfaces of 20° with guiding patterns was further investigated. When the cells migrate and encounter a step, they will migrate in four different directions: moving up the slope to the top of the step, moving down the slope to the bottom of the step, moving along the side of the step, or moving back. To classify the cell migration directions, the edge of the sloped step was used as the reference. Cell migration trajectories were obtained by tracking the movements of individual cells from the time-lapse images. Cells that reached the edges of the sloped steps were analyzed. Cell movements were categorized into four groups: moving up the slope, defined as migration from the bottom to the top of the steps; moving down the slope, defined as migration from the top to the bottom of the steps; moving sideways, defined as migration along the sloped sidewalls; and moving back, defined as cells that approached the edges of the sloped steps but reversed migration direction and moved away from the slopes.

**Figure 3:**
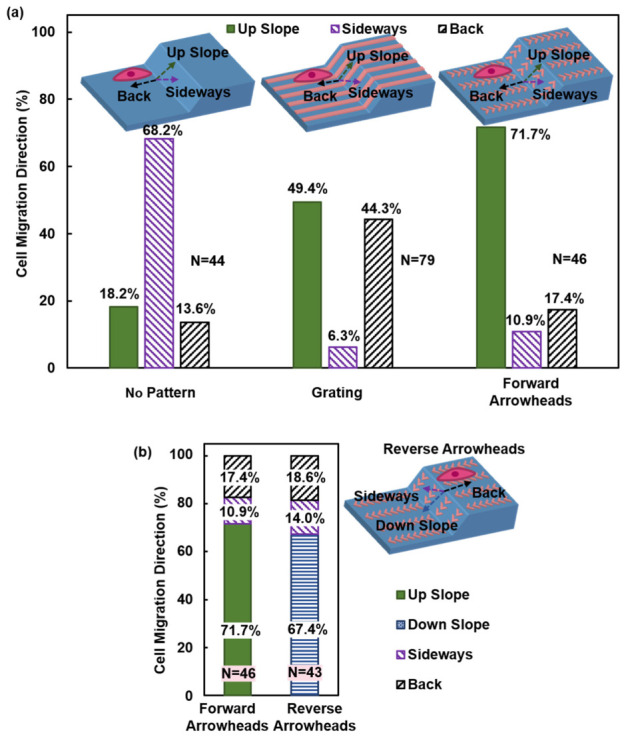
MC3T3-E1 cell migration directions along 20° sloped steps. (**a**) Cell migration directions including cells moving up the slope, sideways, and back after they reached the bottom of sloped steps with no pattern, with grating, and with forward arrowheads. (**b**) Cell migration directions for forward and reverse arrowheads which depended on orientation of arrowheads along the slope.

Figure 3 shows the probabilities of MC3T3-E1 cell migration directions along 20° sloped steps with different surface patterns. Two sets of comparisons were conducted: the first set compared no pattern, grating, and forward arrowheads with tips of arrowheads pointing up from the bottom to the top of the steps, while the second set examined the influence of arrowhead orientation by comparing forward and reverse arrowheads. Reverse arrowheads had tips of arrowheads pointing down from the top to the bottom of the steps. The forward and reverse arrowhead patterns were designed to analyze whether the direction of cell migration across sloped steps can be reversibly controlled by the orientation of patterns with asymmetrical topographies. Specifically, the forward arrowheads guided the cells to migrate along the direction of the arrowhead tips whereas the reverse arrowheads guided the cells to migrate in the opposite direction. With both types of arrowhead patterns, cell migration along the sloped steps can be controlled precisely from the bottom to the top of the steps or changed to the reversed direction down the steps.

In the first group, cells on surfaces without patterns showed predominantly sideways migration (68.2%), with only 18.2% moving up the slope and 13.6% migrating backward, as shown in Figure 3a. On gratings, more cells migrated up the sloped steps (49.4%), a large fraction (44.3%) moved backward, and only 6.3% migrated sideways. In contrast, forward arrowheads further enhanced directional guidance, with 71.7% of cells moving up the slope, while only 10.9% moved sideways and 17.4% migrated backward. In the second group, the role of arrowhead tip orientation was assessed by comparing forward and reverse arrowheads, as shown in Figure 3b. While forward arrowheads facilitated 71.7% of cells in moving up the slope steps, reverse arrowheads promoted migration down the sloped steps, with 67.4% of cells moving down the steps, 14.0% sideways, and 18.6% backward.

**Figure 4:**
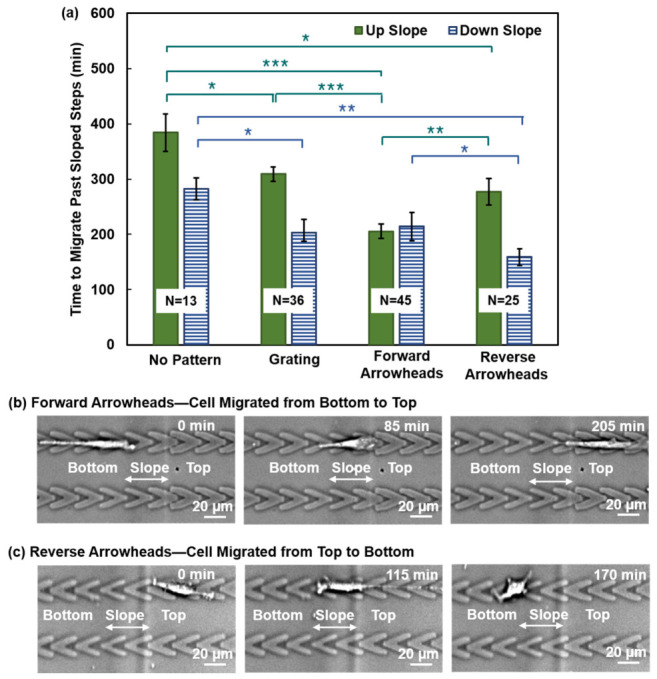
Time for MC3T3-E1 cells to migrate past sloped sidewalls. (**a**) Time to migrate up slope and down slope over 20° sloped steps. One-way ANOVA and Tukey’s post hoc test were applied to analyze statistical significance with * *p* < 0.05, ** *p* < 0.01, and *** *p* < 0.001. Optical micrographs of cell migration (**b**) up slope over step with forward arrowheads and (**c**) down slope over step with reverse arrowheads.

To further investigate the migration speed of MC3T3-E1 cells on different surfaces, the time required for cells to migrate pass the 20° sloped steps was calculated, as shown in Figure 4. Firstly, the migration times of MC3T3-E1 cells on different surfaces were compared for cell movement up the steps, as shown in Figure 4a. On surfaces without patterns, cells required 384.4 ± 33.8 min to migrate up the sloped steps, while gratings reduced this time to 309.4 ± 13.4 min. Reverse arrowheads required 277.3 ± 23.8 min. Forward arrowheads accelerated cell migration up the steps, taking only 205.5 ± 17.3 min, indicating that forward arrowheads provide the most efficient guidance for cell migration up the sloped steps. Next, times to migrate down the sloped steps were analyzed. Cells on surfaces without patterns required 282.5 ± 20.1 min, and gratings reduced this to 202.7 ± 14.9 min. Forward arrowheads resulted in a migration time of 214.2 ± 10.8 min, whereas reverse arrowheads facilitated the fastest migration time of 158.8 ± 15.4 min down the sloped steps. Grating pattern provides symmetrical bidirectional guidance, allowing protrusions to extend from both sides of the elongated cells along the grating orientation. Consequently, cells migrate bidirectionally and frequently change their migration directions, which makes them less efficient in migrating in a single direction. Although reverse arrowheads do not favor the initiation of upslope cell migration, their asymmetrical geometry promotes persistent migration once cell protrusions up the sloped steps are formed. As a result, cells that started migration up the sloped steps could traverse the steps more rapidly than cells on gratings.

For Figure 4, we analyzed a large number of cells (N > 65) in the control group on platforms with sloped steps but no guiding pattern. Out of this large population of analyzed cells over multiple runs and platforms, only a few cells (N = 13 out of 65 cells) were able to successfully migrate over the sloped steps in the absence of the guidance cues. Therefore, the small percentage of the cells that were able to migrate over the steps without guiding patterns further highlights the critical role of patterned guidance cues in promoting cellular migration over sloped steps. The cell seeding density of 1.5 × 10^4^ cells/cm^2^ provided a large number of single-cell trajectories for statistical analysis. Cell interaction with other cells would result in contact inhibition of locomotion and consequently alter their migration behaviors which would be dominated by cell–cell interactions. In this study, the focus is on single-cell migration guidance and control, where cell–cell interactions were excluded.

Representative optical micrographs of MC3T3-E1 cell migration up and down the sloped steps on surfaces patterned with forward and reverse arrowheads are shown in Figure 4b,c. Figure 4b shows cell migration up the step and the corresponding time. After a cell on a forward arrowhead pattern reached the bottom of a sloped step, it typically passed the slope in 85 min, and the entire cell body arrived at the top of the step at 205 min. In contrast, Figure 4c illustrates cell migration down the step, where a typical cell on a reverse arrowhead pattern passed the slope in 115 min, and the entire cell body arrived at the bottom at 170 min. These images highlight how arrowhead orientation guides unidirectional cell migration along sloped steps.

### 3.3. Cell Morphology and Surface Contact on TiO_x_ Guidance Patterns During Migration over Sloped Steps

**Figure 5:**
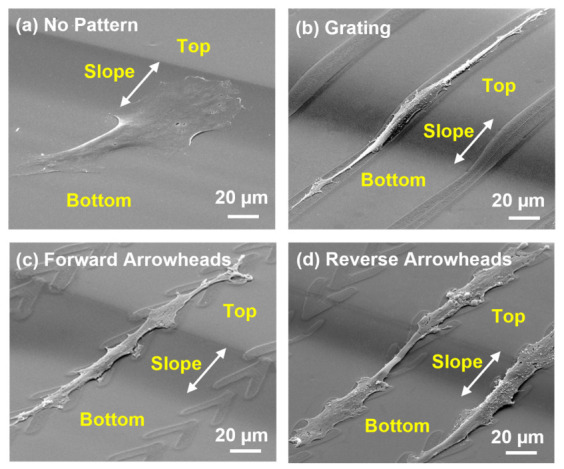
Micrographs of MC3T3-E1 cells migrating over sloped steps with (**a**) no guiding pattern, (**b**) grating, (**c**) forward arrowheads, and (**d**) reverse arrowheads.

Cell morphology was investigated to better understand the mechanisms underlying unidirectional cell migration on patterned TiO_x_ arrowheads across sloped steps. The cellular responses to the thin TiO_x_ patterns were examined using micrographs of MC3T3-E1 cells migrating over sloped steps, as shown in Figure 5. Notably, cells exhibited a preference for spreading and adhering onto the TiO_x_ patterned surfaces as compared to the PDMS surface. As shown in Figure 5a, cells on the PDMS sloped surface without a guiding pattern exhibited spreading of the cell membrane over a large area. On the other hand, MC3T3-E1 cells on patterned TiO_x_ surfaces aligned along the guiding structures. Figure 5b shows that cells on the patterned TiO_x_ grating had a symmetrical shape over the step, likely responsible for the bidirectional movement of the cells along grating. However, cells on the patterned TiO_x_ arrowheads demonstrated alignment along the arrowheads, typically with the leading region of the cell protruding toward the arrowhead tip directions as shown in Figure 5c,d. Lamellipodia were asymmetrically distributed with a larger cell membrane area near the leading region.

### 3.4. Effects of Angle of Sloped Steps on Cell Migration

**Figure 6:**
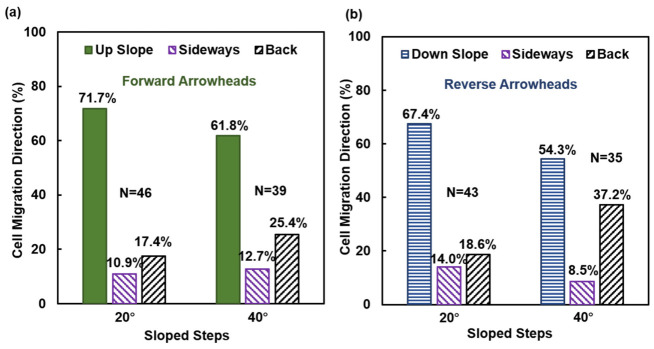
Probability of MC3T3-E1 cell migration on sloped PDMS platforms. Cell migration directions were analyzed for 20° and 40° sloped steps with (**a**) forward and (**b**) reverse arrowheads. Cell migration directions were categorized as up slope, down slope, sideways, and back, highlighting differences for steps with different slope angles.

In order to gain deeper insights into step slope angle-dependent migration behavior, platforms with slope angles of 20° and 40° were prepared. Figure 6 shows the probability of MC3T3-E1 cell migration on sloped PDMS platforms with forward and reverse TiO_x_ arrowheads on sloped steps with slope angles of 20° and 40°. Cell migration directions included up the step, down the step, sideways along the slope, and back after reaching the edge of the step. With forward arrowheads on 20° slope steps as shown in Figure 6a, 71.7% of cells migrated up the 20° steps, while 10.9% moved sideways and 17.4% migrated backward. When the slope angle increased to 40°, upward migration decreased to 61.8%, accompanied by slightly higher proportions of sideways (12.7%) and backward (25.4%) movement. In contrast, with reverse arrowheads on 20° slope steps as shown in Figure 6b, 67.4% of cells migrated down the 20° steps, with 14.0% moving sideways and 18.6% moving backward. As the slope angle increased to 40°, downward migration further declined to 54.3%, whereas backward migration increased markedly to 37.2% and sideways migration slightly decreased to 8.5%. The results showed that the orientation of TiO_x_ arrowhead patterns determined the cell migration directionality and could be used to guide the cells up or down the sloped steps. This serves as a general design principle in which geometrical asymmetry of the guiding patterns could be used to regulate cell migration behaviors on biointerfaces.

Time for MC3T3-E1 cells to migrate past sloped steps with slope angles of 20° and 40° was further studied. Supplementary Figure S2a shows the cell migration time of MC3T3-E1 cells on 20° and 40° sloped steps as cells migrated up the sloped steps. For steps with 20° slope, cells on surfaces without patterns required 384.4 ± 33.8 min to migrate up the slope, whereas gratings significantly reduced this time to 309.4 ± 13.4 min. Reverse arrowheads further shortened the time to 277.3 ± 23.8 min. Notably, forward arrowhead patterns provided the most efficient migration guidance up the steps, with cells requiring only 205.5 ± 17.3 min to completely migrate from the bottom to the top of steps. For steps with a slope of 40°, similar trends were observed, although the overall passing times were shorter. This is because the step height was kept constant at 10 µm; therefore, a larger slope angle reduced the distance on the sloped surface. Consequently, despite having lower cell migration speeds on the steeper slopes, cells traversing a shorter distance over the 40° sloped steps had shorter passing times. Cells on bare surfaces required 258.6 ± 20.7 min, while gratings reduced the time to 235.0 ± 15.5 min. Forward arrowheads again facilitated the fastest migration up the step at 169.2 ± 17.0 min, whereas reverse arrowheads resulted in longer times of 225.0 ± 18.9 min, suggesting forward arrowheads are most effective to enhance cell migration up the sloped steps.

Supplementary Figure S2b presents cell migration times of MC3T3-E1 cells down the 20° and 40° sloped steps. For steps with 20° slope, cells on bare surfaces required 282.5 ± 20.1 min to migrate down the slope, while gratings substantially reduced this time to 202.7 ± 14.9 min. Forward arrowheads yielded a migration time down the slope of 214.2 ± 10.8 min, whereas reverse arrowheads enabled the fastest migration, with cells requiring only 158.8 ± 15.4 min. For steps with 40° slope, the overall times were shorter or similar compared to steps with 20° slope. Cells on bare surfaces required 188.0 ± 34.6 min, and gratings resulted in 207.0 ± 18.6 min. Forward arrowheads reduced the time to 160.0 ± 10.8 min, while reverse arrowheads again facilitated the fastest down slope migration at 155.0 ± 15.9 min, confirming reverse arrowheads provided the most effective guidance for cells to migrate down the sloped steps.

Supplementary Figure S3 presents the migration speed of MC3T3-E1 cells on 40° sloped steps with different guiding patterns. On surfaces without patterns, cells migrated at an average speed of 0.05 μm/min up the slope and 0.07 μm/min down the slope. Gratings slightly increased the speed to 0.06 μm/min for moving up the slope, while the speed for moving down the slope remained at 0.07 μm/min. Forward arrowheads further enhanced cell migration speed, with both moving up and down the slope reaching 0.08 μm/min. In contrast, reverse arrowheads preferentially facilitated cells migrating down the sloped steps, with a migration speed of 0.06 μm/min up the slope and a higher speed of 0.09 μm/min down the slope.

## 4. Discussion

These findings suggest that the patterned 10 nm thick TiO_x_ on flat PDMS platforms with a 0° slope angle can significantly influence the behavior of MC3T3-E1 cells by regulating their alignment, migration and morphology. A thin layer of patterned TiO_x_ provided stronger control over cell migration than physical contact guidance alone. However, gratings were not sufficient to direct migration toward a single direction, highlighting the need for more effective geometries for unidirectional guidance. To address this limitation, arrowhead-shaped TiO_x_ patterns were fabricated on PDMS substrates with sloped steps of 10 μm height, to guide cell migration along sloped steps. Patterned TiO_x_ arrowhead surfaces enhanced directional cell migration, with forward arrowheads being the most effective in providing guidance up the sloped steps. The improvement can be attributed to the anisotropic geometry of the arrowheads, which provided stronger unidirectional cues than gratings, allowing cells to align their morphology and focal adhesions (FAs) along the tips of the arrowheads and thereby increasing the probability of cells moving up the slope steps in the same direction as the tips of arrowheads [33]. This observation is also consistent with our previous findings that patterned ultrathin TiO_x_ arrowheads could direct cell migration along the tips of the arrowheads by inducing asymmetrical FA formation. Preferential localization of FAs at the arrowhead tips enhanced cell adhesion and protrusion along the direction of the arrowhead tips, thereby promoting unidirectional cell migration.

In addition, forward arrowhead tips were pointed up from the bottom to the top of the steps, facilitating the extension of lamellipodia and filopodia upward along the slope. This allows cells to reorganize their cytoskeleton optimally for upward movement over the steps [34]. Time to migrate up and down the steps depended on whether cell migration direction was aligned with or against the direction of the arrowhead tips. On forward arrowheads, the difference between migration time up and down the sloped steps was small. In contrast, reverse arrowheads showed a pronounced disparity, with migration time down the steps much faster than up the steps. Depending on whether cell migration is aligned or opposed to the tip orientation, this guidance either facilitates or impedes cell movement, producing a pronounced directional effect on both migration speed and trajectory.

These observations also demonstrate that forward arrowheads facilitate efficient cell migration up the steps, whereas reverse arrowheads preferentially guide cell migration down the sloped steps, corroborating with the cell migration direction results observed in Figure 3. Arrowhead patterns with tip alignment with or against the cell migration direction strongly influence both the migration direction and speed of cells migrating over sloped steps. This phenomenon is consistent with previous reports that showed aligned ratchet-shaped fibronectin patches inducing biased long-term motion through asymmetric protrusion formation [35,36]. Therefore, the ultrathin TiO_x_ arrowhead patterns on PDMS platforms with sloped steps enabled control of cell migration directions over steps, which provided a guideline for designing engineered platforms to direct cell movements.

On PDMS sloped steps with no patterns as shown in Supplementary Video SV1(a), cells migrated up and down the sloped steps in a random, non-directional manner. Under grating guidance as shown in Supplementary Video SV1(b), cells exhibited elongated morphologies, and most of them showed bidirectional movements along the gratings as they migrated up and down the steps. With forward arrowhead patterns as shown in Supplementary Video SV1(c), the majority of cells migrated up the sloped steps along the direction of the arrowhead tips, whereas with reverse arrowhead patterns as shown in Supplementary Video SV1(d), most cells descended down the steps along the arrowhead tips. As cells migrated up the sloped steps, cellular protrusions sensed the guiding patterns and established good contact over the sloped surface. In contrast, when cells migrated down the sloped steps, cellular protrusions continuously probed the guiding patterns, and once they contacted the guidance cues at the bottom of the step, cells often had less surface contact on the slope, and they rapidly retracted their trailing regions and migrated to the bottom of the step. In general, cells migrating up the sloped step exhibited better surface contact, which increased cell adhesion, whereas cells migrating down the step showed less surface contact, thereby facilitating faster movement [3,37,38].

The cell migration guidance by the arrowhead patterns became less effective for larger slope angles. This observation is consistent with previous studies showing that, on convex cylindrical surfaces, increasing curvature inhibits cell migration in directions perpendicular to the cylinder’s axis [22,25,28,31,39,40]. Notably, sideways migration remained relatively low across all conditions (14%), indicating that TiO_x_ arrowhead patterns effectively constrained cells from lateral spreading and maintained alignment with the guidance pattern orientation. Collectively, these findings indicate that larger slope angles resulted in less effective cell migration guidance for cells to migrate across sloped steps. The forward arrowheads enhanced migration speed up the slope, whereas reverse arrowheads preferentially increased speed down the slope. These findings confirm that the orientation of arrowhead tips can modulate both the speed and directionality of cell migration over sloped steps based on the geometrical asymmetry of the guiding patterns.

In our previous study, PDMS micropost sensing platforms with nanopillars, silicon oxide, and titanium oxide on top of the microposts were developed to monitor the dynamic cell traction force during migration [4]. The relationships between various platform surfaces, migration behaviors, and cell traction forces were analyzed. Compared with PDMS surfaces, cells on titanium oxide surfaces showed reduced mobility and less elongation, which is related to the smaller exerted traction force. In the future, traction force monitoring for cell migration over sloped steps will be carried out to study the biomechanical aspects of cell migration over a physical incline.

## 5. Conclusions

In this study, 10 nm thick TiO_x_ cell migration guidance structures were patterned on flat PDMS platforms with a 0° slope angle to investigate cell movement on planar surfaces as a control group. In the absence of guiding patterns, cells exhibited random migration. When guided by TiO_x_ gratings, cells displayed pronounced bidirectional migration along the grating orientation. On the other hand, cells on arrowhead patterns migrated primarily along the arrowhead tip orientation. The asymmetrical geometry of the arrowheads introduced unidirectional cues that enable cells to reorganize their morphology and their migration towards the direction of the arrowhead tips. Cell migration on sloped steps of 20° with guiding patterns was further investigated. On unpatterned sloped steps, cells migrated randomly with a strong tendency for sideways movement. Introducing ultrathin TiO_x_ gratings on the sloped surface significantly enhanced migration directionality, guiding cells to migrate over the sloped steps. However, gratings mainly supported bidirectional movement, with limited control over the preferred migration direction up or down the steps. In contrast, forward arrowheads markedly increased the probability of cells migrating up the slope to 71.7% while minimizing sideways and backward movements, whereas reverse arrowheads promoted cells migrating down the slope to 67.4%. Overall, forward arrowheads promote cell migration up the steps, while reverse arrowheads guide migration down the steps.

Migration time across the sloped steps of 20° further demonstrated that arrowhead orientation governs not only migration direction but also migration speed. Compared with cells on other patterns, cells on forward arrowheads exhibited the shortest migration time of 205.5 ± 17.3 min up the steps, while those on reverse arrowheads enabled the fastest migration time of 158.8 ± 15.4 min down the steps. Thus, the arrowhead tip orientation can facilitate cell migration along tip direction. To further investigate the effects of step slope angle on cell migration behaviors, platforms with a slope angle of 40° were developed. Increasing the slope angle from 20° to 40° reduced both the probability of up slope migration and the migration speed on the sloped surfaces. However, because steeper steps resulted in shorter distance along the sloped surfaces when the step height was kept to a constant of 10 µm, the total step-crossing times were shorter. Nevertheless, the arrowhead patterns maintained strong directional guidance even at a steeper slope angle of 40°, demonstrating their robustness as topographical cues. The slope angles of 20° and 40° were selected to represent moderate and steeper steps, enabling systematic evaluation of cell migration under different degrees of incline in ECM. The slope widths were designed to match the characteristic length of spread MC3T3-E1 cells, allowing cells to simultaneously interact with the bottom, sidewall, and top of the sloped steps during migration. A continuous slope geometry was not included as this study was focused on understanding how cells migrated across steps vertically in 3D microenvironments. In addition, asymmetrical guiding patterns were developed to regulate directional cell migration. Overall, ultrathin patterned TiO_x_ arrowheads enable effective cell migration guidance across sloped steps, with the migration direction depending on the orientation of the arrowhead tips. The results demonstrate that geometrical asymmetry can be applied to regulate directional cell migration across sloped steps for topography-guided cell migration in 3D microenvironments. The ultimate aim of this study is to provide mechanistic and design insights for controlling cell migration in complex 3D microenvironments.

## Data Availability

The original contributions presented in this study are included in the article/Supplementary Materials; further inquiries can be directed to the corresponding author.

## Supplementary Materials

The following supporting information can be downloaded at: https://www.mdpi.com/article/10.3390/jfb17070323/s1. Supplementary Figure S1. (a) MC3T3-E1 cell migration speed in x- and y-directions to show migration directionality. (b) Migration speed of cells on platforms with no pattern, grating, and arrowheads. All platforms were flat surfaces without step. One-way ANOVA and Tukey’s post hoc test were applied to analyze statistical significance with ** *p* < 0.01 and *** *p* < 0.001; Supplementary Figure S2. Time for MC3T3-E1 cells to migrate pass sloped steps with slope angles of 20° and 40°. (a) Cells migrating from bottom to top of sloped steps. (b) Cells migrating from top to bottom of sloped steps; Supplementary Figure S3. Migration speed of MC3T3-E1 cells over 40° sloped steps on different platforms. One-way ANOVA and Tukey’s post hoc test were applied to analyze statistical significance with * *p* < 0.05 and ** *p* < 0.01; Supplementary Movie SV1. MC3T3-E1 cells migrating on different PDMS platforms with sloped steps. (a) No pattern. (b) Gratings. (c) Forward arrowheads. (d) Reverse arrowheads.
