# Supplementary material for: Guiding of Cell Migration over Sloped Steps Using TiOx Arrowhead Patterns
Source: J Funct Biomater. 2026 Jul 5;17(7):323. doi: 10.3390/jfb17070323 (PMC13413221; doi:10.3390/jfb17070323)
Supplement: Supplementary file 1 [file jfb-17-00323-s001.zip › jfb-4319262-Supplementary Materials.pdf]

Electronic Supplementary Information (ESI)

Guiding of Cell Migration over Sloped Steps Using TiO_x Arrowhead Patterns

Yijun Cheng[1,2,3], Chang Liu[1,2], and Stella W. Pang[1,2,3]*

[1]Department of Electrical Engineering, City University of Hong Kong, Kowloon, Hong Kong

[2]Centre for Biosystems, Neuroscience, and Nanotechnology, City University of Hong Kong, Kowloon, Hong Kong

[3]State Key Laboratory of Terahertz and Millimeter Waves, City University of Hong Kong, Kowloon, Hong Kong, China

*Corresponding Author: pang@cityu.edu.hk

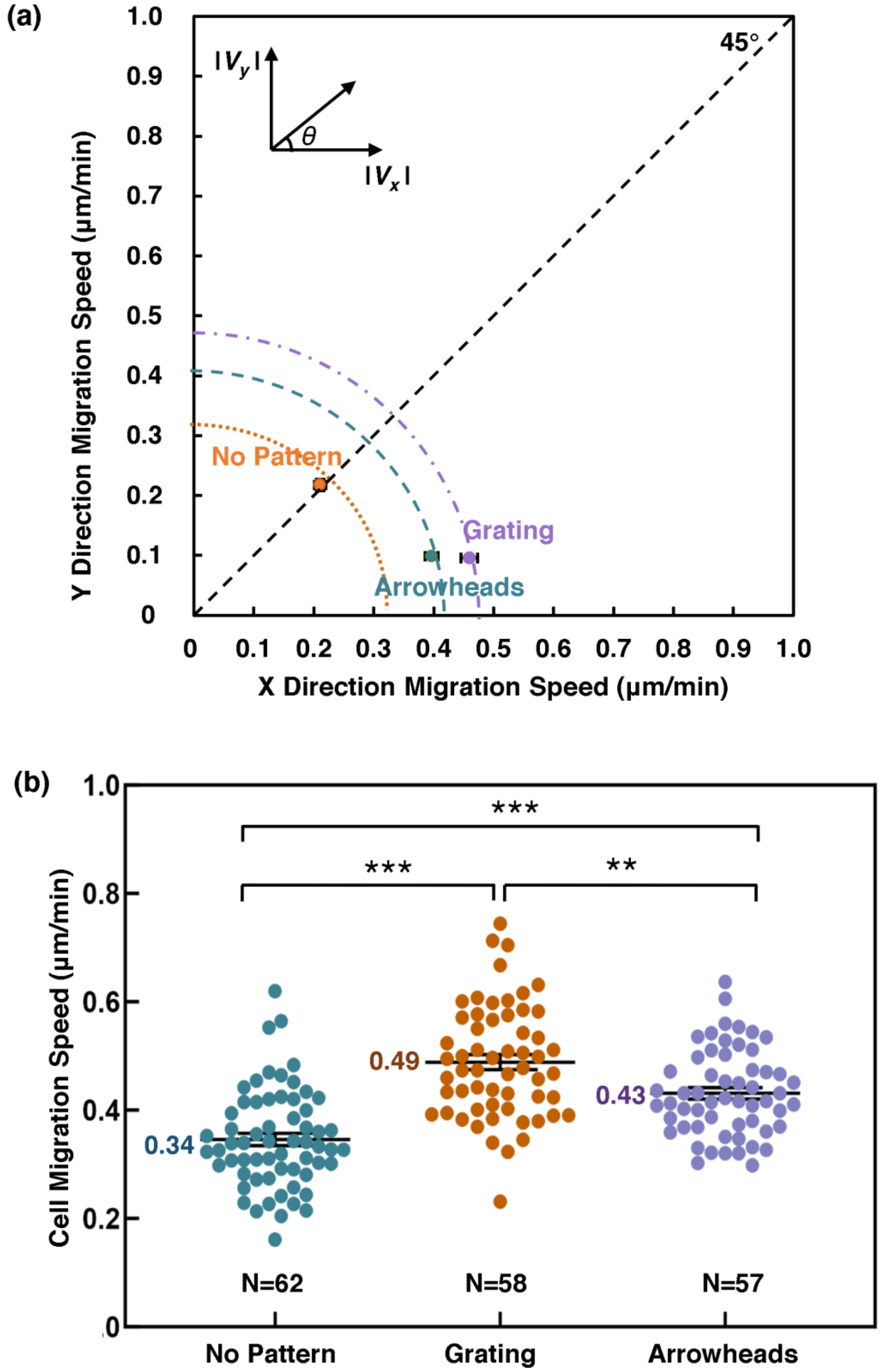

Supplementary Figure S1. (a) MC3T3-E1 cell migration speed in x- and y-directions to show migration directionality. (b) Migration speed of cells on platforms with no pattern, grating, and arrowheads. All platforms were flat surfaces without step. One-way ANOVA and Tukey's post hoc test were applied to analyze statistical significance with **p <0.01 and ***p <0.001.

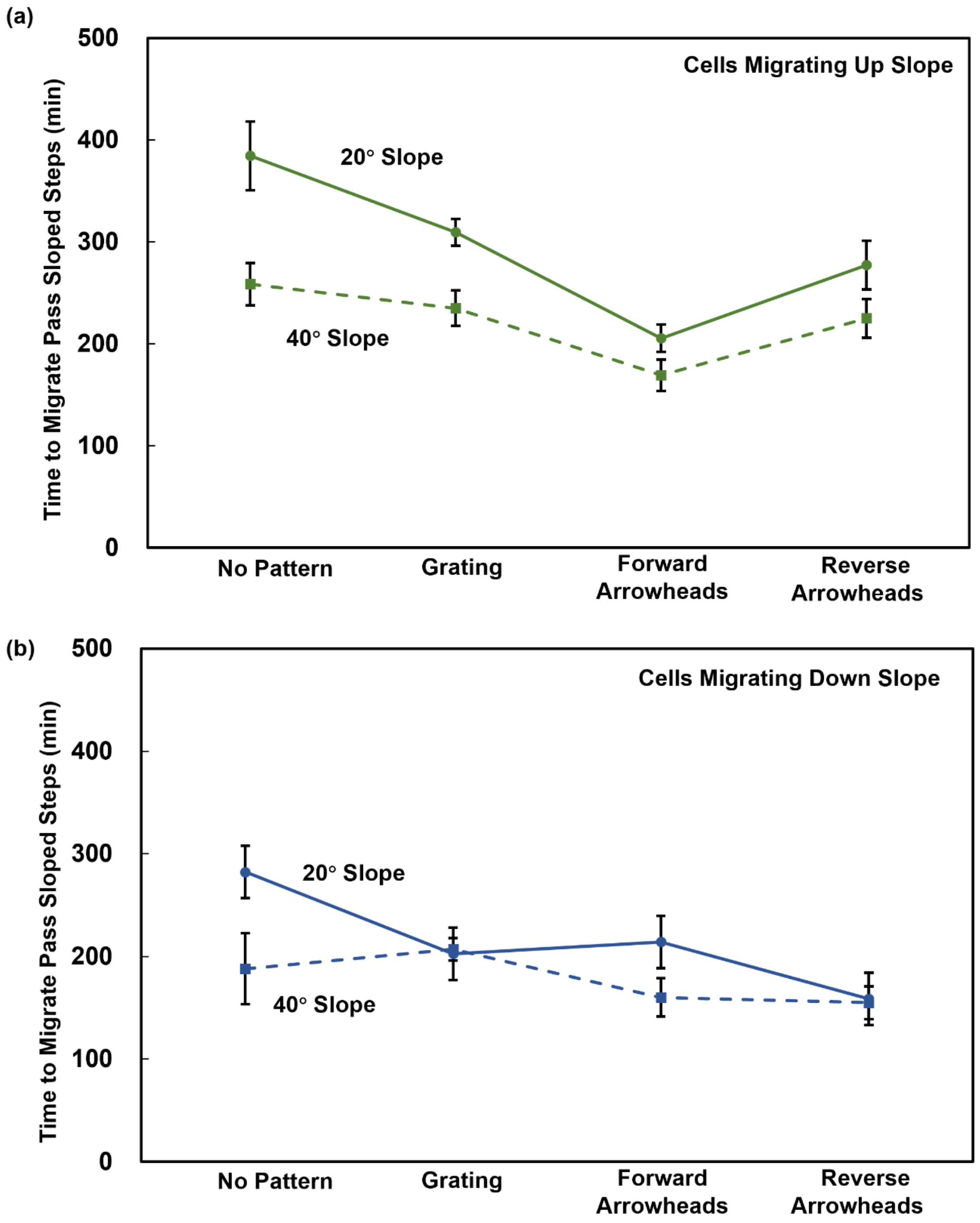

Supplementary Figure S2. Time for MC3T3-E1 cells to migrate pass sloped steps with slope angles of 20° and 40°. (a) Cells migrating from bottom to top of sloped steps. (b) Cells migrating from top to bottom of sloped steps.

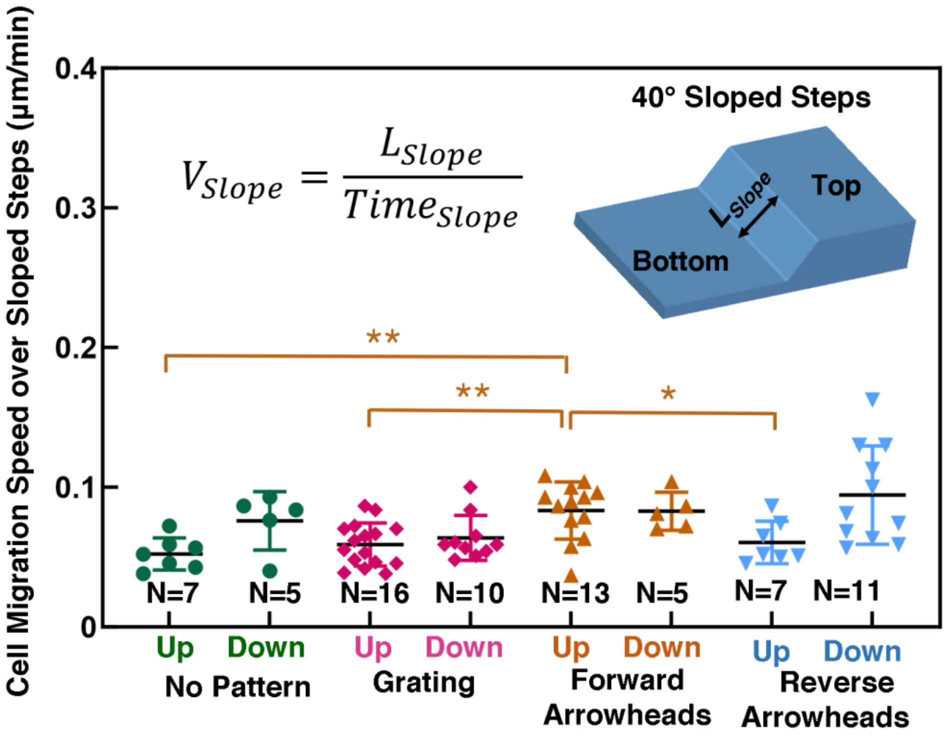

Supplementary Figure S3. Migration speed of MC3T3-E1 cells over 40° sloped steps on different platforms. One-way ANOVA and Tukey's post hoc test were applied to analyze statistical significance with *p <0.05 and **p <0.01.

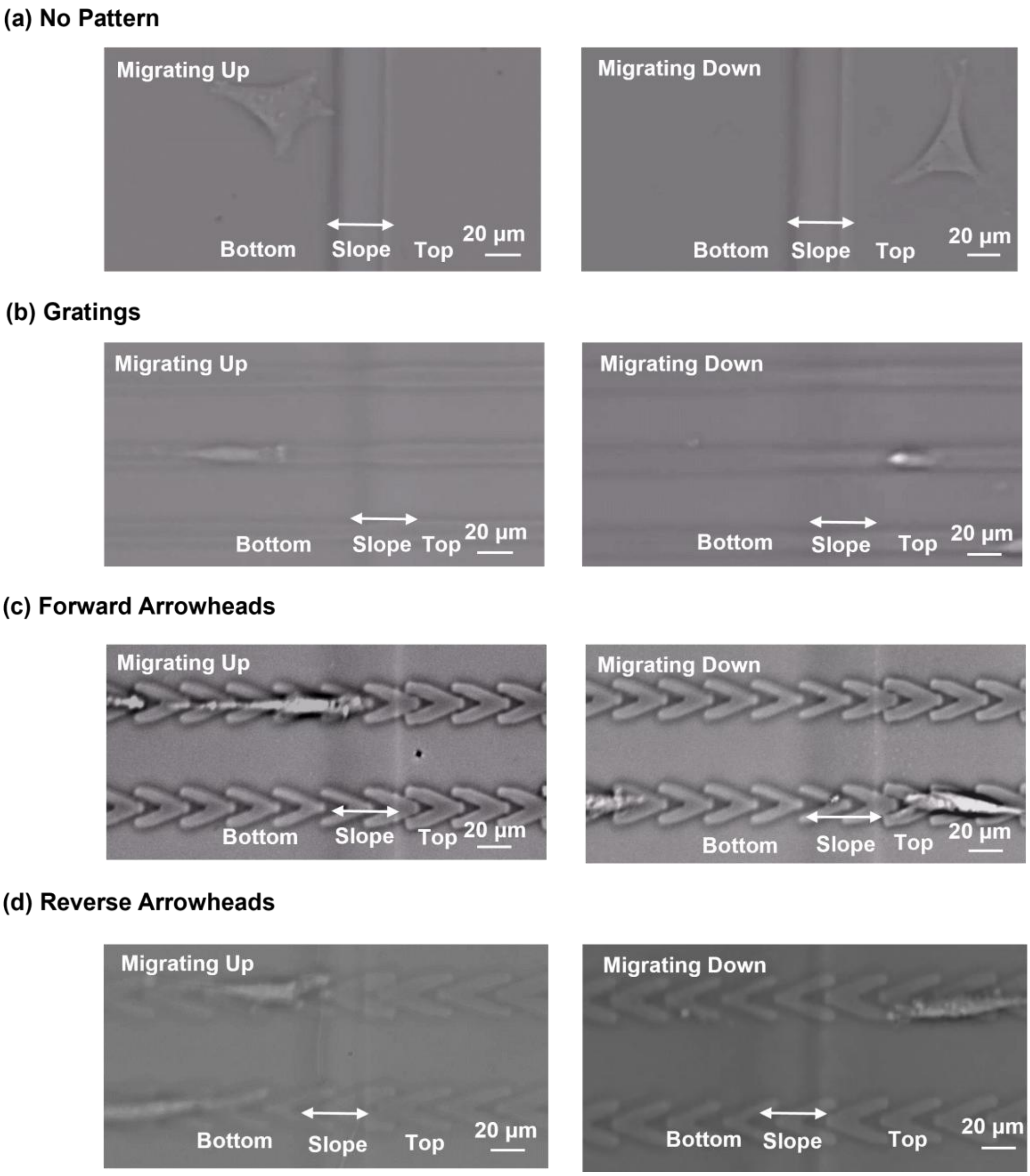

Supplementary Movie SV1. MC3T3-E1 cells migrating on different PDMS platforms with sloped steps. (a) No pattern. (b) Gratings. (c) Forward arrowheads. (d) Reverse arrowheads.
